# Complete genome sequence of *Chryseobacterium* sp. strain KCF3-3, isolated from the body surface of channel catfish, *Ictalurus punctatus*

**DOI:** 10.1128/mra.01058-24

**Published:** 2024-12-20

**Authors:** Miho Kojima, Kaho Tobioka, Mika Okazaki, Kiyonobu Yokota, Dien Arista Anggorowati, Hajime Nakatani, Katsutoshi Hori, Yutaka Tamaru, Fumiyoshi Okazaki

**Affiliations:** 1 Department of Life Sciences, Graduate School of Bioresources, Mie University, Tsu, Mie, Japan; 2 Research Center for Marine and Land Bioindustry, National Research and Innovation Agency (BRIN), North Lombok, West Nusa Tenggara, Indonesia; 3 Department of Biomolecular Engineering, Graduate School of Engineering, Nagoya University, Nagoya, Aichi, Japan; 4 Section of Soft and Functional Materials, Tohoku University Green Crosstech Research Center, Sendai, Japan; 5 Department of Molecular Bioengineering, Graduate School of Engineering, Tohoku University, Sendai, Japan; Montana State University, Bozeman, Montana, USA

**Keywords:** channel catfish, *Ictalurus punctatus*, mucus, *Chryseobacterium*

## Abstract

Here, we report the complete genome sequence of *Chryseobacterium* sp. strain KCF3-3, isolated from the body surface of channel catfish, *Ictalurus punctatus*. The *de novo* assembly revealed a chromosome size of 5,623,437 bp with an estimated 4,939 open reading frames.

## ANNOUNCEMENT

Members of the genus *Chryseobacterium* are rod-shaped, non-spore-forming, nonmotile Gram-negative bacteria that are found in diverse environments (1). Here, we report the complete genome sequence of *Chryseobacterium* sp. KCF3-3 to enhance our understanding of its ecological diversity. We isolated the strain from the body surface of channel catfish, *Ictalurus punctatus* in 2019 (Table 1). Mucus samples were collected from four *I. punctatus* specimens and cultured on Reasoner’s 2A agar plates at 25°C for 24 h to obtain the isolates. After three rounds of purification, isolates were identified by molecular phylogenetic analysis based on the 16S rRNA gene sequences. The primers used for amplification were 27 F-W (5′-AGRGTTTGATCMTGGCTCAG-3′) and 1,492 R-W (5′-GGYTACCTTGTTACGACTT-3′). The neighbor-joining methods were used to analyze the genetic relationship (2). Consequently, the strain KCF3-3, which is classified within the genus *Chryseobacterium*, was obtained.

**TABLE 1:** Genomic features of *Chryseobacterium* sp. strain KCF3-3

Parameter	
Description of location	
Location	Lake Kasumigaura, Ibaraki, Japan
Time	26 October 2019
Type	Body surface of *I. punctatus*
Geographic coordinates	36°05'39.0"N/140°24'09.3"E
Sequencing statistics	
Number of raw reads	18,579
Mean length	13,306
Total bases	247,205,453
Number of Filtlong filtered reads	16,184
Genome statistics	
Assembly size (bp)	5,623,437
Number of contigs	1
GC content (%)	36.0
Genome coverage	44.0
Number of 5S rRNA	6
Number of 16S rRNA	6
Number of 23S rRNA	6
Number of tRNAs	87
Total number of coding sequence	4,939
Completeness (%)	99.99
Contamination (%)	0.08
Data accession	
BioProject	PRJDB18362
BioSample	SAMD00797993
GenBank accession No.	AP035792

The genomic DNA of strain KCF3-3 was extracted using NucleoBond HMW DNA (Macherey-Nagel) and further purified using DNA Clean Beads (MGI Tech Co., Ltd.) according to the manufacturer’s protocol. The concentration of the DNA solution was measured using the QuantiFluor dsDNA system on a Quantus Fluorometer (Promega). The DNA was fragmented to approximately 10–20 kbp using g-TUBE (Covaris) by centrifuging three times at 2,100 × *g*. Fragmented lengths were confirmed by measuring with a 5200 Fragment Analyzer (Agilent Technologies). The DNA library was prepared using the SMRTbell Express Template Prep Kit 2.0 according to the Procedure and Checklist instructions (Part number 101–730-400 ver. 06). Polymerase complexes were prepared by Revio Polymerase kit (PacBio), and sequencing was performed using Revio (PacBio) by Bioengineering Lab. Co., Ltd. in 2024. SMRT Link (ver. 13.0.0.207600) was used to remove overhang adaptors, and consensus sequence reads with an average quality value of less than 20 per read were removed. Filtlong (ver. 0.2.1) was used to eliminate reads shorter than 1,000 bases and yielded 18,579 reads with an N_50_ value of 14,599 bp. *De novo* assembly was performed using Flye (ver. 2.9.2-b1786) (3, 4), and Bandage (ver. 0.8.1) (5) and CheckM2 (ver. 1.0.1) (6) were used for quality assessment of the assembled genome. Flye detects overlapping contig ends as circular candidates and automatically circularizes them (3). Genome annotation was performed using the Prokka software (ver. 1.14.6) (7).

**Fig 1:**
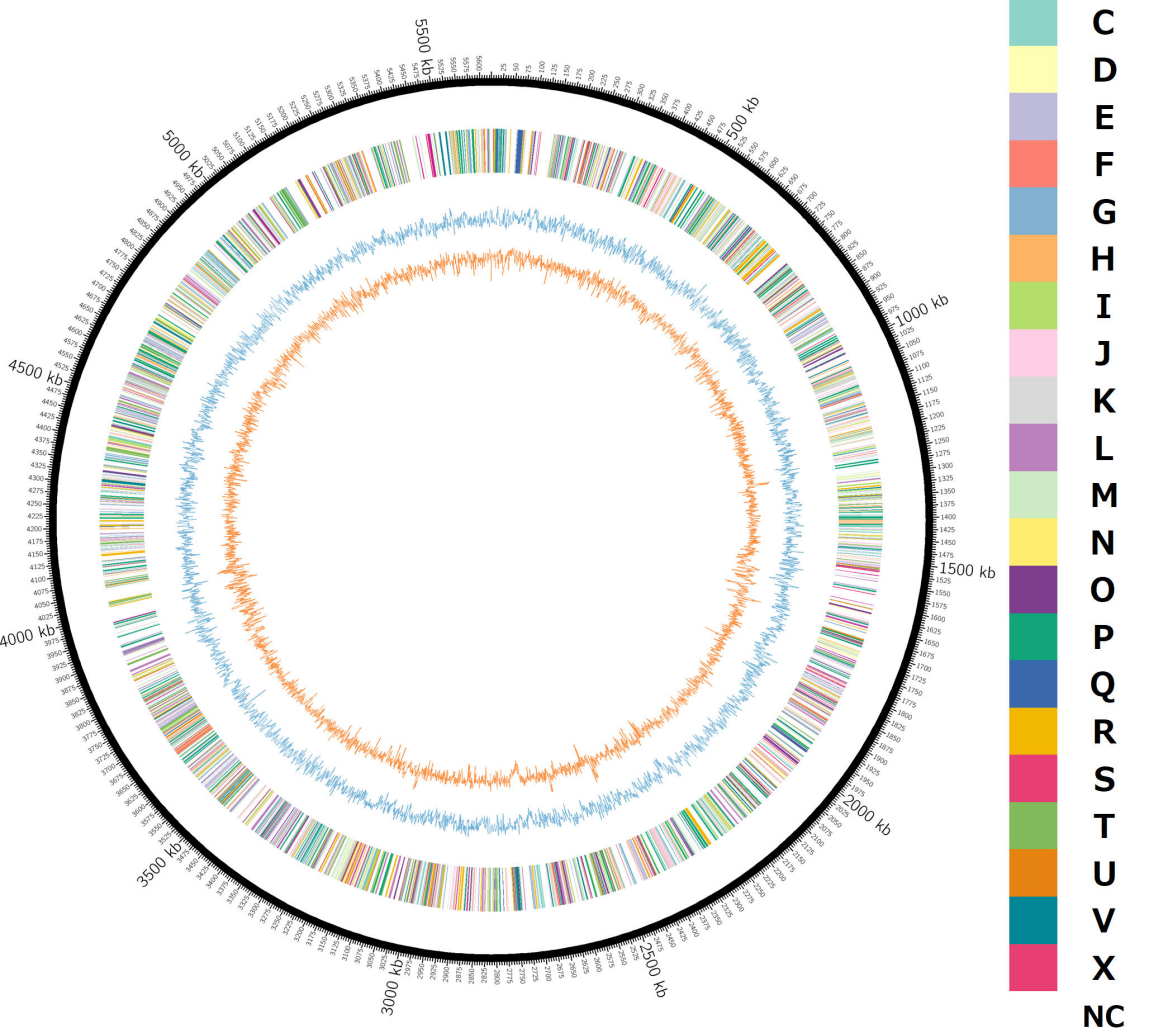
Circular representation of the genome of *Chryseobacterium* sp. strain KCF3-3. From outer circle to inner circle: color of inferred COG categories, GC skew (blue), and GC contents (orange). NC in COG color code indicates no classified category.

The chromosome size of the strain KCF3-3 was 5,623,437 bp, and the contig was a single circularized genome. The genome coverage was 44.0×, and the GC content was 36.0%. The genome contains 4,939 coding sequences. Prokka predicted 105 RNA genes (87 tRNA genes and 18 rRNA genes). These results and other genomic information are shown in Table 1. Clusters of Orthologous Groups (COG) categories were estimated using DFAST (https://dfast.ddbj.nig.ac.jp) (8, 9), and GC contents and GCskew were calculated using GCcalc (https://github.com/WenchaoLin/GCcalc). These results were visualized using Circos (ver. 0.69) (10) (Fig. 1). All software used default parameters, unless otherwise specified.

## Data Availability

The whole-genome shotgun project for the strain KCF3-3 has been deposited in GenBank under accession number AP035792. The raw reads and raw sequencing data are available under BioProject accession number PRJDB18362 and BioSample accession number SAMD00797993.
